# Wild *Allium * species (Alliaceae) used in folk medicine of Tajikistan and Uzbekistan

**DOI:** 10.1186/1746-4269-2-18

**Published:** 2006-04-03

**Authors:** Michael Keusgen, Reinhard M Fritsch, Hikmat Hisoriev, Parvina A Kurbonova, Furkat O Khassanov

**Affiliations:** 1 Philipps-Universität Marburg, Institut für Pharmazeutische Chemie, Marbacher Weg 6, D-35032, Marburg; 2 Institut für Pflanzengenetik und Kulturpflanzenforschung, Corrensstraße 3, D-06466, Gatersleben; 3 Botanical Institute of the Tajik Academy of Sciences, Karamov Street 27, 734017 Dushanbe, Republic of Tajikistan; 4 Scientific Centre of Plant Production "Botanika" of the Uzbek Academy of Sciences, F. Khodzhaev Street 32, 700143 Tashkent, Republic of Uzbekistan

## Abstract

**Background:**

Hitherto available sources from literature mentioned several wild growing *Allium *species as "edible" or "medicinally used" but without any further specification.

**Methods:**

New data were gained during recent research missions: *Allium *plants were collected and shown to the local population which was asked for names and usage of these plants.

**Results:**

Information was collected about current medical applications of sixteen wild species, nine of which belong to different sections of *Allium *subgenus *Melanocrommyum*. These plants are used against headache, cold, and stomach problems, and are mostly applied fresh or after boiling.

**Conclusion:**

Close taxonomic relatives of the common onion were used similar to cultivated onion species, but medical use like garlic was mostly reported for species taxonomically not related to garlic.

## Background

About 200 different *Allium *species were reported for the mountainous regions of Middle and South-West Asia [1]. The use of especially tasteful and curative members of this family has a long tradition in several Asian populations with apparently deep historical roots. This assumption might be illustrated by the fact that also people living in urban areas since generations know how to prepare special dishes from particular plants. They are buying the required plant material at local markets where it is sold by rural providers which collected it in the wild. Also ancient reports about common onion (*Allium cepa *L.) and garlic (*A. sativum *L.) are coming out of this area bearing antique civilizations. While wild ancestors of the worldwide most important cultivated *Allium *species could not be identified yet without doubt, genetically and phylogenetically most closely related plants are exclusively distributed in this part of Asia [2].

**Figure 1:**
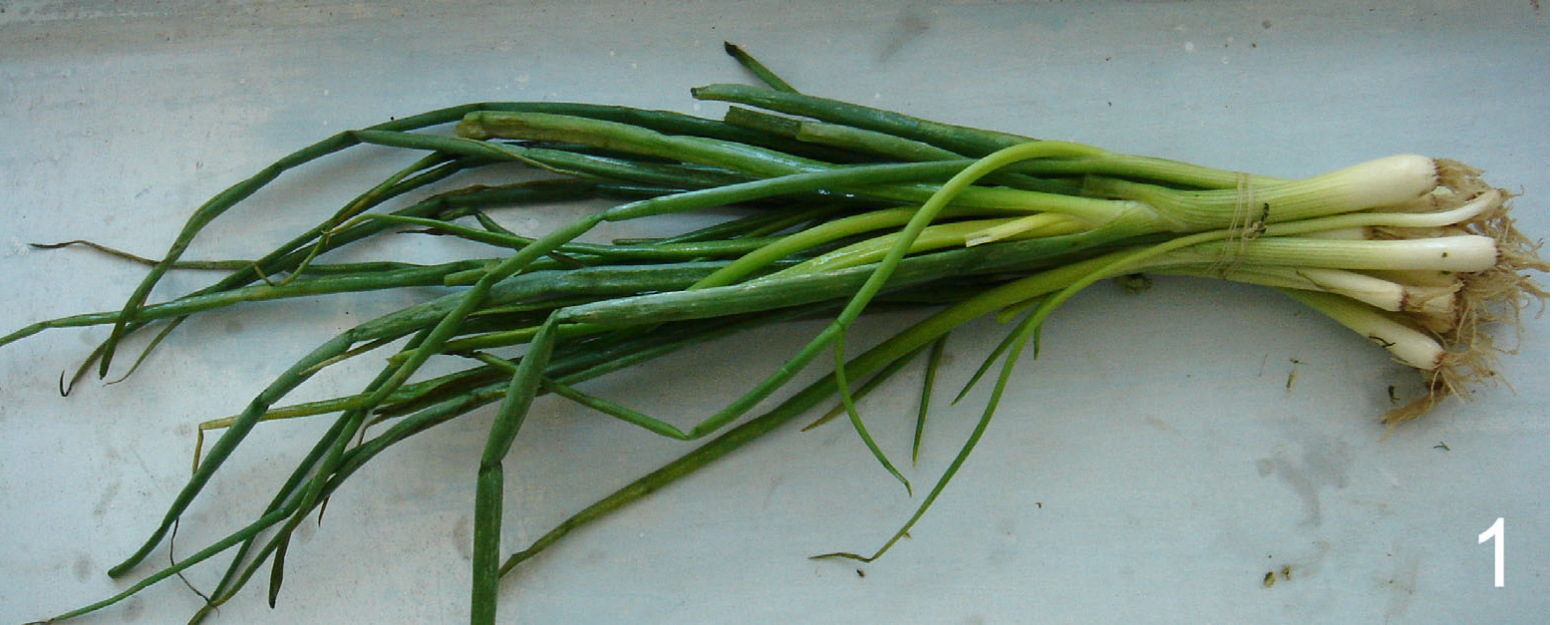
**Sample figure title. **Young plants of common onion are frequently eaten as greens and sold on nearly every local market (photo taken in Dushanbe, Tajikistan).

The economically most important *Allium *crop species (common onion and garlic) are worldwide used as spices, vegetables, and medicinal plants. Traditionally, they play a very important role in the daily diet also in Asia. Here they can be seen under cultivation in every home garden. This holds also true for the territories of Tajikistan and Uzbekistan where young fresh plants (Fig. 1) and dry bulbs are offered at every local market and are generally also used as medicinal plants. A rather recent review [3] described the wide spectrum of medical properties of both crop species.

Besides theses two cultivated species, also a number of wild species are collected and eaten by the local population [4] but separate references to medical applications are rarely given [5,6]. Most literature sources mention only that particular species were eaten, emit a characteristic smell like onion or garlic, or are used as spice or medicinal plants without presenting further details. Sometimes the use of wild *Allium *species is described as 'used as common onion'. Again, this description is very diffuse und means, that some parts of the plant can be either used as vegetable, spice, or herbal drug. Also a differentiation between true vegetable plants and spicy vegetables (*e.g.*, leek *A. porrum *L. in Europe and North America) is rarely given.

**Figure 2:**
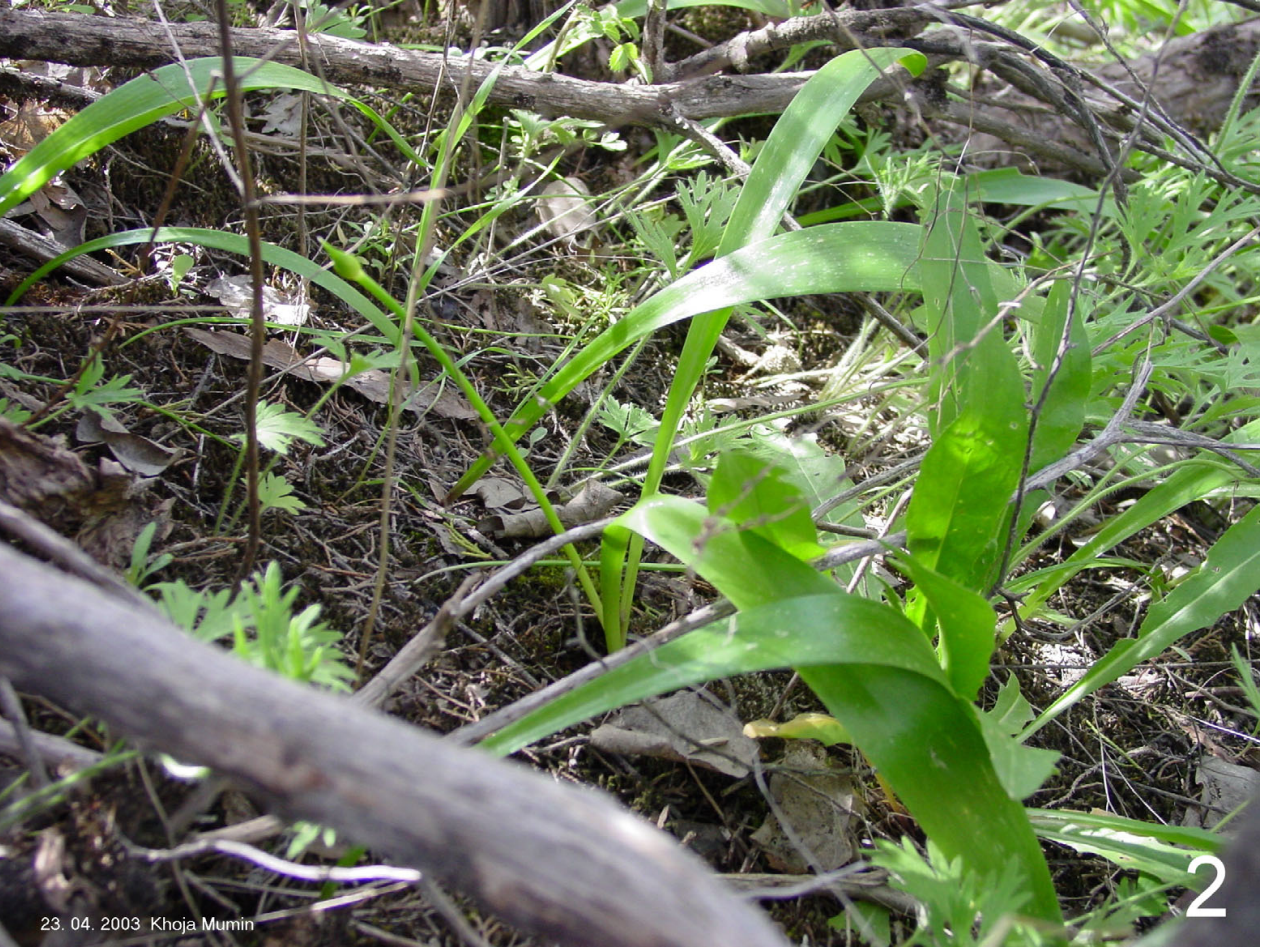
**Another sample figure title. ***Allium rosenbachianum*: leaves of this size are collected for consumption (photo taken in South Tajikistan).

**Figure 3:**
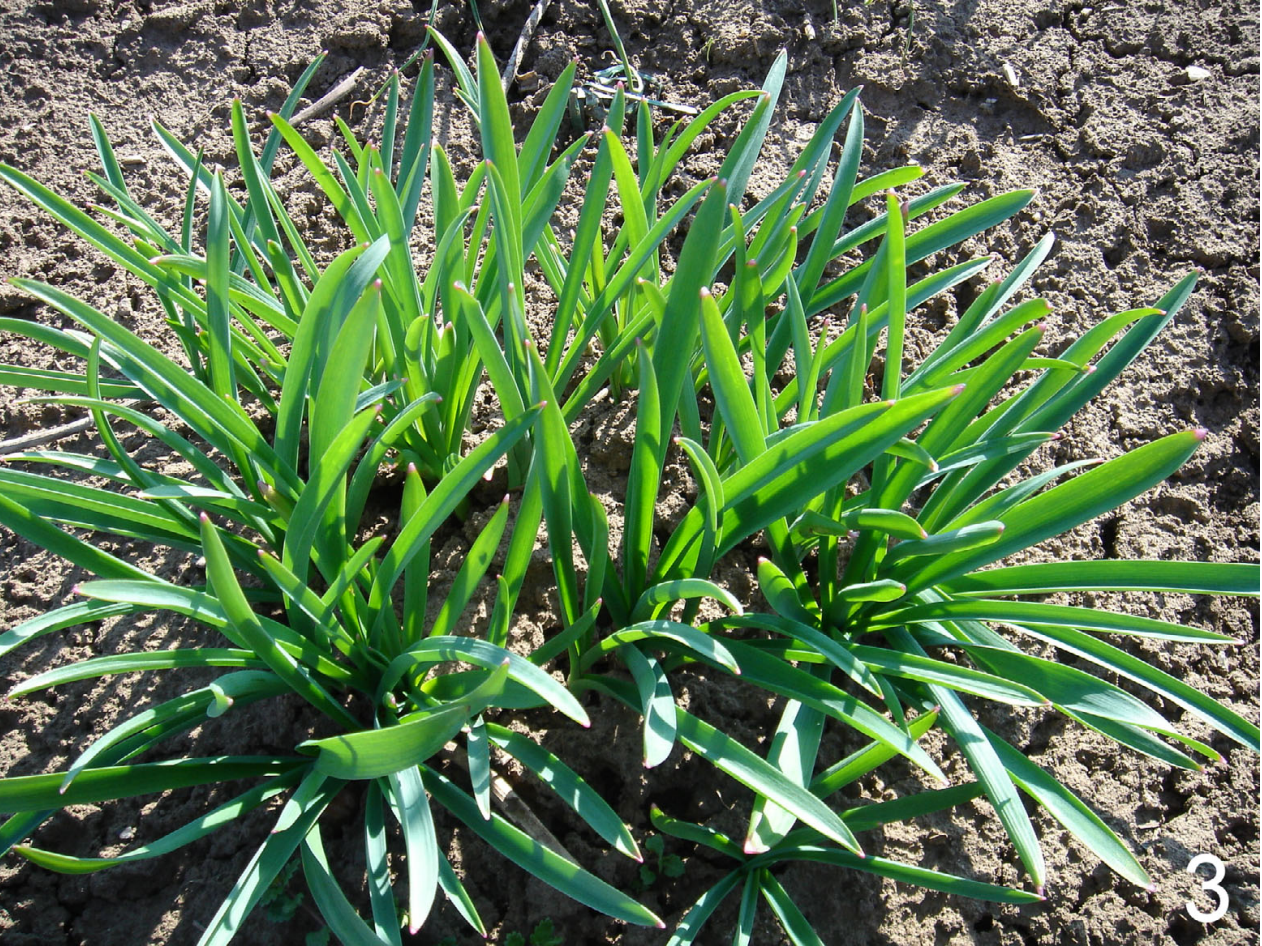
**Another sample figure title. ***Allium rosenorum*: leaves of the given size are collected for consumption (photo taken from the reference collection).

Detailed information is not even supplied for widely used species. An exception is the dissertation of Umarov [7]. In Tajikistan and in some parts of neighbouring countries where related tribes settled, leaves of *A. rosenbachianum *auct. – this name is used in some scientific literature for *A. rosenbachianum *(Fig. 2) in a strict sense as well as for *A. rosenorum *(Fig. 3) – are extensively used for traditional dishes. According to literature [8,9], this species is applied as spice and as vegetable as ingredient of soups. In the 1980ies during botanical expeditions, one of the authors (RMF) was repeatedly informed by local people that these leaves, which do not own any special taste, are often collected and eaten because consumption "refreshes the body after the winter period". Thus, the reported use as spice needs confirmation or exclusion.

Another example for confusing reports in literature is related to *A. stipitatum*. Information about the use of this species is in accordance for both countries that only young bulbs were pickled and highly esteemed as spicy vegetable [6,10]. A medicinal use was not reported yet but could not be surely excluded. Wild species listed above can be obtained at local markets throughout.

Sensoric properties as well as health benefits of *Allium *species can be related to a broad variety of secondary metabolites of these plants. Most important are sulphur compounds, which are typically alk(en)yl sulphoxides of the amino acid cysteine. If plant material is damaged, the enzyme alliinase comes in contact with cysteine sulphoxides like methiin, alliin, isoalliin, and propiin, and a broad variety of biologically active compounds is formed [3]. Variations in the combination and concentration of enzymatically formed sulphur compounds result in species- and group-specific taste and smell. Also the health benefit of *Allium *species can be deduced from sulphur components. If the concentration of sulphur compounds is rather low, species were often used as vegetable giving a very mild taste after cocking. Species with a high sulphur content like garlic were mainly used as spice and/or medicinal plant.

Besides sulphur compounds, also saponins were reported for a number of *Allium *species and may also contribute to the health benefits of these plants [3]. Sugars, typically fructose, are responsible for the sweet taste of a number of *Allium *species, mostly related to *A. cepa*. Moreover, phenolic compounds were detected in this genus and must be considered as bioactive compounds, too.

Not one of these publications mentioned in Table 1 gives any reference about the scientific background of the data and presented vouchers (photographs, herbarium specimens, or other stored material) or described identification of the plants. Therefore, a research project was initiated for collecting new data, which follows another strategy to minimize error sources. The currently available results are presented below.

**Table 1:** Medical application of wild *Allium *species in Tajikistan and Uzbekistan

Local Name	Application	Region of Usage	Nationality	Accessions	Source
**subgenus *Rhizirideum *(G. Don ex Koch) Wendelbo section *Cepa *(Mill.) Prokh.**					
*Allium oschaninii *O. Fedt.					
piozi kuhi	Leaves and bulbs are used against fever, headache, kidney problems, and stomach-ache.	Central Tajikistan, Darvaz mountain range	Tajik	6080, 6100	12
*Allium pskemense *B. Fedt.					
	The bulb juice is used as a medicine.	Uzbekistan: West Tianshan mountain range	(not reported)	(no vouchers given)	10
tog pioz	The whole fresh plants and bulb juice are used against stomach problems, tuberculosis, and, like boiled bulbs, against strong cold. Young leaves and stems or boiled and smashed bulbs are applied to wounds and against skin diseases.	Uzbekistan: West Tianshan mountain range	Uzbek	4142	14
	The bulb juice is used against tuberculosis and strong cold.	Central Asia	(not reported)	(no vouchers given)	15
**subgenus *Rhizirideum *section *Campanulata *Kamelin**					
*Allium barsczewskii *Lipsky					
sir	Bulbs and pounded leaves are applied at the head against colds and flue, headache, fever, and toothache. Seeds are eaten with bread in order to increase appetite.	Central Tajikistan: Vakhsh mountain range	Tajik	6105	14
*Allium jodanthum *Vved.					
piozi dashti	Fried bulbs are laid onto the face in case of nerve problems; fresh bulbs are used against toothache.	Tajikistan: Panj Karatau Mts.	Tajik	6040	12
yovoj pioz	Leaves and bulbs without stems are used against toothache and mumps, alcoholic extracts for disinfections of wounds.	Uzbekistan: West Tianshan Mts.	Uzbek	4141, 4188	14
**subgenus *Rhizirideum *section *Oreiprason *F. Herm.**					
*Allium talassicum *Regel					
	Any medicinal usage denied.	Uzbekistan: West Tianshan mountain range	Uzbek	4217	14
	Used in folk's medicine (not specified).	Uzbekistan: West Tianshan mountain range	(not reported)	(no vouchers given)	10
dasht-niyaz	Bulbs are used against scurvy and for health recovery.	Central Asia	(not reported)	(no vouchers given)	15
	Bulbs are used against scurvy and for health recovery.	Tajikistan	(not reported)	(no vouchers given)	16
**subgenus *Rhizirideum *section *Schoenoprasum *Dumort.**					
*Allium fedschenkoanum *Regel					
qamch pioz	Dried plants are cut and burnt, and the smoke is inhaled against cold.	Central Tajikistan: Darvaz mountain range	Tajik	6197	12
**subgenus *Allium *section *Allium***					
*****Allium filidens *Regel					
piozi diona	Bulbs are applied against headache.	Tajikistan: Darvaz mountain range	Tajik	6069	12
**subgenus *Melanocrommyum *(Webb et Berth.) Rouy **str.					
*Allium chitralicum *Wang et Tang s.					
siri kuhi	Smashed bulbs are used against sense of fear, and the whole plant for the national dish 'atolla'.	Tajikistan: West Pamir	Tajik	6097	12
*Allium hissaricum *Vved.					
sir	Fresh or dried leaves are applied against headache and fever.	Central Tajikistan	Tajik	6106	12
*Allium karataviense *Regel					
	Applied as medicine (not specified).	Uzbekistan: West Tianshan mountain range	(not reported)	(no vouchers given)	10
	Applied for quicker healing of wounds.	North Tajikistan	(not reported)	(no vouchers given)	5
	Used against pneumonia and lung problems.	Central Asia	(not reported)	(no vouchers given)	15
*Allium komarowii *Lipsky					
gushi gurgak	Used as an anabolic for horses.	Tajikistan: Hissar mountain range	Tajik	6134	12
khujrak-motor	Leaves and bulbs are fresh used, or cut in pieces and cooked and applied against anaemia and bad circulation.	Uzbekistan: West Hissar mountain range	Uzbek	4170	14
*Allium motor *Kamelin et Levichev					
motor	In spring the leaves are highly esteemed as stuffing for a special variant of the national pie dish 'somsa', medical properties not mentioned.	Uzbekistan: West Tianshan mountain range	(not reported)	(no vouchers given)	10
moj-modor	Young leaves are eaten in soups and 'somsa' which owns a specific activity as tonic.	Uzbekistan: West Tianshan mountain range	Uzbek	4133	14
*Allium rosenbachianum *Regel subsp. *rosenbachianum *and subsp. *kwakense *R.M. Fritsch					
	Young leaves are used as condiment for soups, no medical properties mentioned.	Central Tajikistan	(not reported)	(no vouchers given)	6
gushi buzak	Fresh and dried leaves represent the vegetable part of the national soup dish 'atolla' which is much esteemed as appetizer and general tonic.	Central Tajikistan: Panj Karatau Mts., Darvaz and Vakhsh mountain ranges	Tajik	6050, 6051, 6072, 6078, 6107	12
*Allium rosenorum *R.M. Fritsch (*A. rosenbachianum *auct.)					
siekhalaf, siralaf, shipioz, jorji	Young fresh or dried leaves are used for the national soup dishes 'atolla' and 'oshi sioalaf' which have tonic properties.	Central Tajikistan: Vakhsh and Hissar mountain ranges	Tajik	6109, 6143, 6167	12
siohalaf	Young leaves are used as condiment for soups, no medical properties mentioned.	Central Tajikistan	(not reported)	(no vouchers given)	6
*Allium severtzovioides *R.M. Fritsch					
tosh-motor	Fresh leaves and bulbs without stems are locally applied against stomach and duodenum diseases.	Uzbekistan: West Tianshan mountain range	Uzbek	4140	14
*Allium suworowii *Regel					
	Used in folk's medicine (not specified).	North Tajikistan	(not reported)	(no vouchers given)	5
	Used as a medicine (not specified).	Uzbekistan: West Tianshan mountain range	(not reported)	(no vouchers given)	10
piozi anzur	Decocts of flowers and seeds are applied against headache and cold.	Central Tajikistan: Darvaz mountain range	Tajik	6090	12
niyazi-ansul	Pickled bulbs are eaten against tuberculosis and bronchitis.	Central Asia	(not reported)	(no vouchers given)	15
	Used in folk's medicine against early forms of tuberculosis and bronchitis.	Tajikistan (not specified)	(not reported)	(no vouchers given)	16

## Methods

Information was gained during joined research missions with the local cooperation partners in 2003, 2004, and 2005. In the areas of interest, at first fresh plant material was collected and then shown to the native population of this region (mountainous areas of the Republics of Uzbekistan and Tajikistan, mainly belonging to the Hissar mountain range and the Pamir). Because of strict ethnological rules, only male person were asked, but these often showed plant material to further members of the family, also female persons. People were interviewed in their native language. Because of significant migrations during the time of the former Soviet Union, results were not related to specific ethnological sub-populations. Also, results were not related to the age of interviewed persons, but in most cases they had an age of about 50 to 60 years and were often recommended by rural communities as experts for traditionally used plants. People were asked about the local name and whether they are using these plants, and if so, which part is taken, for what purpose, and how is it prepared and stored.

Afterwards, the presented plant material was transferred to the national living *Allium *collections of Tajikistan in Dushanbe (curator: Prof. Dr. H. Hisoriev, accessions beginning with '6'), or of Uzbekistan in Tashkent (curator: Dr. F. Khassanov, accessions beginning with '4'), resp., for further cultivation, documentation, and taxonomic determination. In Tashkent and Dushanbe also voucher specimens of the accessions are deposited. Duplicates of some accessions were also transferred to the Taxonomic *Allium *Reference Collection of the Institute of Plant Genetics and Crop Plant Research (IPK), Gatersleben, Germany, to be re-determined if necessary. All data concerning collecting, cultivation, and questioning the native population were assembled in an electronic database.

If possible, results from interviews were compared with data available from literature as indicated in Table 1. It must be noticed, that most literature data do lack an unambiguous botanical description of plant material. Also, specific plant parts (leaves, bulbs, stems) used by men were not mentioned in most cases. This lack of former knowledge was closed by the now presented study.

## Results

The obtained data confirm that a remarkable number of wild *Allium *species is collected for consumption by Uzbek and Tajik people. Inside the visited countries, no further differentiation into ethnic groups was done. Several species are only used as vegetable and/or spice plants. They have special importance in rural areas during springtime when vegetables cannot be bought in the shops or are too expensive, and the garden plots do not give yield yet. Such species will be not considered here, because investigations are ongoing. Other wild *Allium *species are eaten due to a special health benefit, or applied in case of medical indications (see Table 1).

## Discussion

**Figure 4:**
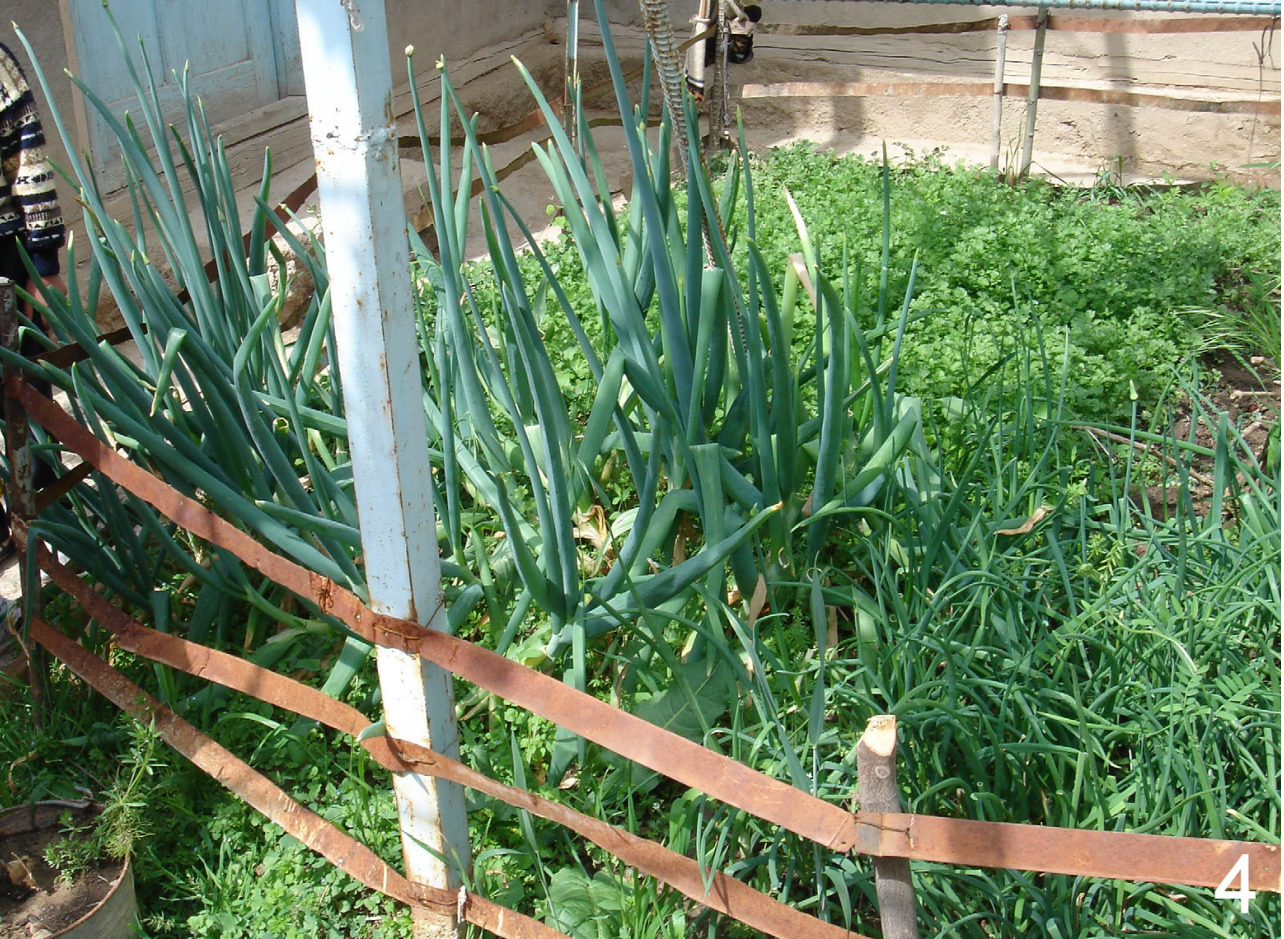
**Another sample figure title. ***Allium pskemense *(left) and common onion (right) are jointly grown in a home garden near Tashkent (Uzbekistan).

Three wild *Allium *species growing in Tajikistan and Uzbekistan (*A. oschaninii*, *A. pskemense*, *A. praemixtum*) are closely related to common onion. They are traditionally collected and used as spice like common onion, but only *A. oschaninii *and *A. pskemense *are also medicinally applied. Thus, we can confirm reports about the use of *A. oschaninii *in Uzbekistan and Tajikistan [4,6,8,10]. We can also verify that *A. pskemense *is sometimes grown in home gardens in Uzbekistan (Fig. 4) for usage like common onion and as medicinal plant.

Although the only species of subgenus *Allium *mentioned in Tab. 1 (*A. filidens*) is taxonomically rather distantly related to garlic, but it is medically used in a similar manner. This kind of medical applications also holds true for two species from the rhizomatous section *Campanulata *and for four species from the bulbous subgenus *Melanocrommyum*. This usage is apparently not strictly related to the content of the cysteine sulphoxide alliin ('mother compound' of the active principle), which is high in garlic, *A. filidens, A. barsczewskii*, and *A. jodanthum*, but very low in the species of subgenus *Melanocrommyum *shown in Tab. 1 [11]. Further studies will show whether other related compounds not determined yet or completely different chemical compounds might be the reason for this usage.

**Figure 5:**
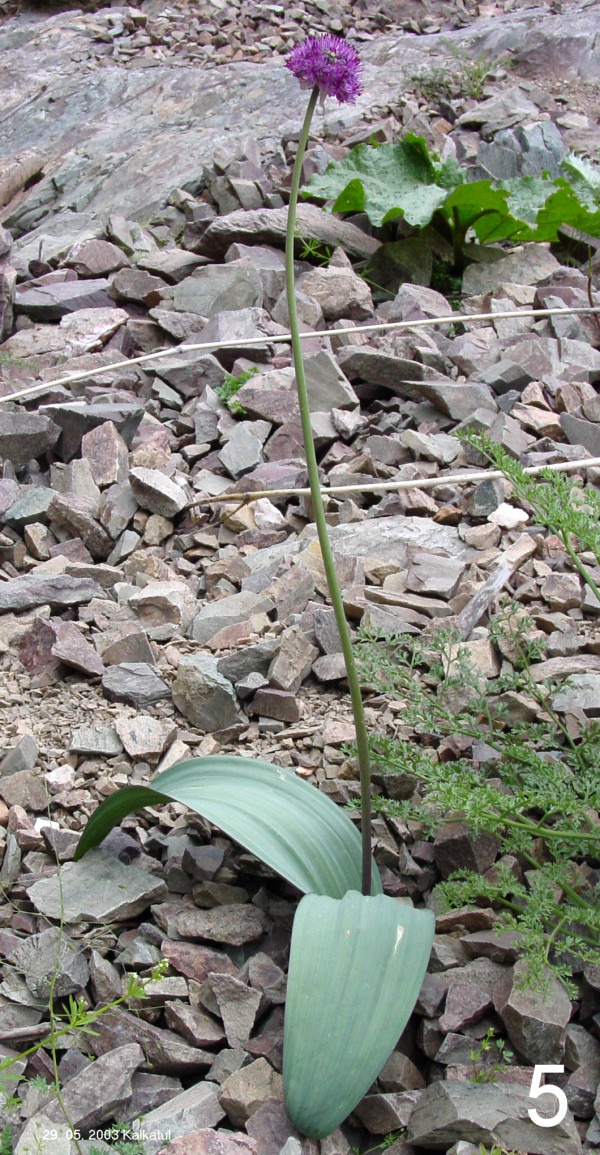
**Another sample figure title. **Flowering plant of *Allium komarowii *in the West Hissar mountain range (Uzbekistan).

*Allium komarowii *(Fig. 5) owns obviously a rather strong medical activity, because it is used as anabolic drug for horses [12]. Also this kind of activity could not be correlated to high cysteine sulphoxide contents [11], but this species contains a conspicuous red dye, which is chemically a sulphurpyrrol [13]. The purified substance showed a strong antioxidative effect but contribution to the health benefit of the entire plant is not clear until now.

Special dishes, which are much esteemed for strong tonic properties, are prepared from the leaves of three species of the subgenus *Melanocrommyum: A. motor*, *A. rosenbachianum*, and *A. rosenorum*. According to our information, these dishes are explicitly consumed because of the tonic property. Also these plants contain the above mentioned red dye, which is regarded as a 'sign of authenticity' when the leaves are collected in April and May. The term 'motor' means 'health', but the local names of the other two species are not related to application. The questioned people always denied that these plants are used as spice.

We were only able to get confirmation that pickled young bulbs of *A. stipitatum *are used as spicy vegetable and not as medicine. However, young bulbs of *A. suworowii *are identically prepared but medicinally used. Both species contain only traces of cysteine sulphoxides [11]. Thus, we must conclude that other substances must be responsible for the differing usages also in this case.

Application against scurvy was only reported in the literature for *A. talassicum *and is not confirmed by own data. It can be assumed that this disease has lost importance because of a better general diet and availability of modern pharmaceuticals for treatment.

The wild *Allium *species listed above are mainly applied fresh or after boiling of mostly dried material (Tab. 1). Fumigation (*A. fedschenkoanum*) and alcoholic extraction (*A. jodanthum*) were only once reported.

In Uzbekistan and Tajikistan, most of the wild species shown in Tab. 1 are not simply used instead of common onion and garlic, but are very specifically applied. This fact may illustrate that collection and application of wild *Allium *species reflects an apparently rather ancient tradition. Several people interviewed during our expeditions underlined that often a certain plant is used by only a part of population living in a specific area, or when commonly applied species are missing in this area. The knowledge of wild *Allium *species was also not observed throughout a local population. Some persons questioned even did know nothing about any wild *Allium *species.

Because the interviews were not always successful and our research missions did not cover the whole territories of both countries, the presented data may only incompletely reflect the existing knowledge in Tajikistan and Uzbekistan. Nevertheless, we were able to find evidence that several *Allium *species mentioned in literature as "edible plants" without detailed specification were utilized for medical applications. Certainly some more edible wild *Allium *species than mentioned in Tab. 1 are also medically applied. Additional investigations seem to be essentially necessary.

## Competing interests

The author(s) declare that they have no competing interests.

## Authors' contributions

All authors took part in the research missions and in collecting and interpretation of the information gained. Additionally, planning and organisation the missions was managed by FOK in Uzbekistan and by HH in Tajikistan. RMF and FOK dealt especially with the botanical and MK with the chemical aspects of research.
